# Medium-term and peri-lockdown course of psychosocial burden during the ongoing COVID-19 pandemic: a longitudinal study on patients with pre-existing mental disorders

**DOI:** 10.1007/s00406-021-01351-y

**Published:** 2021-11-25

**Authors:** Claudia Bartels, Philipp Hessmann, Ulrike Schmidt, Jonathan Vogelgsang, Mirjana Ruhleder, Alexander Kratzenberg, Marit Treptow, Thorgund Reh-Bergen, Mona Abdel-Hamid, Luisa Heß, Miriam Meiser, Jörg Signerski-Krieger, Katrin Radenbach, Sarah Trost, Björn H. Schott, Jens Wiltfang, Claus Wolff-Menzler, Michael Belz

**Affiliations:** 1 Department of Psychiatry and Psychotherapy, University Medical Center Goettingen, von-Siebold-Str. 5, 37075 Goettingen, Germany; 2 Department of Psychiatry and Psychotherapy, University Hospital Bonn, Bonn, Germany; 3 School for Mental Health and Neuroscience, Department of Psychiatry and Neuropsychology, Maastricht University Medical Center, Maastricht, The Netherlands; 4 McLean Hospital, Harvard Medical School, Translational Neuroscience Laboratory, Belmont, MA USA; 5 Department of Psychiatry and Psychotherapy, University of Duisburg-Essen, LVR-Hospital, Essen, Germany; 6 Geriatric Psychiatry, University Department of Geriatric Medicine FELIX PLATTER, Basel, Switzerland; 7 German Center for Neurodegenerative Diseases (DZNE), Goettingen, Germany; 8 Leibniz Institute for Neurobiology, Magdeburg, Germany; 9 Neurosciences and Signaling Group, Institute of Biomedicine (iBiMED), Department of Medical Sciences, University of Aveiro, Aveiro, Portugal

**Keywords:** Coronavirus, SARS-CoV-2, Mental health, Psychosocial stress, Adjustment disorder

## Abstract

While the COVID-19 pandemic continues, patients with pre-existing mental disorders are increasingly recognized as a risk group for adverse outcomes. However, data are conflicting and cover only short time spans so far. Here, we investigate the medium-term and peri-lockdown-related changes of mental health outcomes in such patients in a longitudinal study. A cohort of 159 patients comprising all major mental disorders (ICD-10 F0-F9) were interviewed twice with the Goettingen psychosocial Burden and Symptom Inventory (Goe-BSI) to evaluate psychosocial burden, psychiatric symptoms and resilience at the end of the first (April/May 2020) and the second lockdown in Germany (November/December 2020). For the primary outcome “psychosocial burden” ratings also comprised retrospective pre-pandemic (early 2020) and very early states during the pandemic (March 2020). For all diagnostic groups, psychosocial burden varied significantly over time (*p* < 0.001) with an increase from the pre-pandemic to the initial phase (*p* < 0.001), followed by a steady decrease across both lockdowns, normalizing in November/December 2020. Female gender, high adjustment disorder symptom load at baseline and psychiatric comorbidities were risk factors for higher levels and an unfavorable course of psychosocial burden. Most psychiatric symptoms changed minimally, while resilience decreased over time (*p* = 0.044 and *p* = 0.037). The longitudinal course of psychosocial burden indicates an initial stress response, followed by a return to pre-pandemic levels even under recurrent lockdown conditions, mimicking symptoms of an adjustment disorder. Strategies for proactive, specific and continuous treatment have to address resilience capacities before their depletion in the pandemic aftermath, especially for patients with additional risk factors.

**Supplementary Information:**

The online version contains supplementary material available at 10.1007/s00406-021-01351-y.

## Introduction

As a prolonged global crisis, the continuing COVID-19 pandemic and the associated lockdown measures severely and persistently challenge mental health worldwide. A subsequent increase of mental health problems had been expected [1], and accordingly, a growing body of evidence points to an already elevated prevalence of mental disorders [2–8]. Considerable concern is also raised about a further rise of mental health problems in the long term [9, 10]. Amongst other risk groups and based on the diathesis-stress model as a theoretical framework, patients with pre-existing mental disorders (past or current) were determined as a particularly vulnerable group (e.g. [1, 11]). Such patients, in addition to pre-existing risk factors (mental disorder as diathesis), may be more adversely affected by current environmental stressors (pandemic and related measures) than healthy individuals.

In general, individuals with premorbid mental disorders seem to be predisposed to strongly respond to critical and stressful life events (e.g., [12]). Related to the current pandemic, they were found to exhibit higher COVID-19 infection rates [11, 13], an increased risk of adverse outcomes from COVID-19 [14–16], or both [13, 17]. Moreover, they have been suggested to be particularly susceptible for worsening of their mental health condition in response to pandemic-related stressors, in terms of an exacerbation or relapse of psychopathology or de novo onset of psychiatric symptoms.

Unsurprisingly, in most cross-sectional comparisons, patients with pre-existing mental disorders showed higher levels of symptoms than patients without such disorders. Likewise, previous mental illness was identified as a predictor for worsening in mental health status [18, 19]. Most studies to date are limited to the very early phase of the pandemic in spring 2020. Longitudinal empirical data are now steadily accumulating, but have thus far yielded conflicting results. While some studies support the hypothesis of a particular vulnerability of these patients showing an increase or at least a lesser decrease in symptomatology during the pandemic [20–22], several studies challenge the assumption of a more severe course with unfavorable mental health outcomes in patients with pre-existing mental disorders. Such, samples of these patients were reported to experience only a minor increase [23], no change or even a decrease of symptoms [24–27]. Also, the longitudinal investigation of specific diagnostic subgroups revealed mixed results with increasing symptoms in patients with eating disorders [28–30], decreasing symptoms in adolescents with attention deficit hyperactivity disorder [31] or in individuals with bipolar disorder [22], and differential effects, e.g. on patients with eating [32] or alcohol use disorders [33].

Overall, accumulating empirical evidence suggests that an initially higher symptom load of patients with pre-existing mental disorders does not necessarily outlast the pandemic course but may be stable or even decrease over time (medium- to long-term course). A recent systematic review and meta-analysis of 65 longitudinal cohort studies with data prior to and during the pandemic (until mid-2020) even concluded that the subgroup of patients with pre-existing mental disorders (25 studies) showed no significant change in mental symptoms [34]. However, longitudinal data available so far mainly refers to the first lockdown (very short-term course) and rarely covers periods in summer 2020, i.e., shortly after the easing of the first lockdown measures (short-term course). Therefore, longitudinal peri-pandemic assessment of symptom load in patients with pre-existing mental disorders should cover longer time spans. Building on our previous findings on psychosocial burden of patients in the early weeks of the pandemic during the first lockdown in Germany (April/May 2020 [35]), we here aimed to longitudinally monitor the subsequent development of symptom load in the same patient cohort. The present study represents a follow-up assessment performed during the second lockdown in November/December 2020 intended to evaluate the longitudinal impact of the pandemic by addressing the following questions: (1) How does the ongoing pandemic influence the peri-pandemic and medium-term course of psychosocial burden? (2) Are there additional risk factors possibly contributing to a worsening of mental health outcomes and a more severe course of psychosocial burden? (3) How does the ongoing pandemic influence symptoms of adjustment disorder and general psychiatric symptoms, as well as resilience?

## Material and methods

Study details (eligibility criteria, study procedures) have recently been described in detail [35]. For the purpose of intelligibility, we recapitulate major key points mainly referring to the follow-up assessment.

### Study sample

Patients were eligible if they (1) were aged ≥ 18 years, (2) had been treated previously in the Department of Psychiatry and Psychotherapy at the University Medical Center Goettingen, Germany between 10/2019 to 03/2020, (3) had not been hospitalized at inclusion, (4) were capable of giving informed consent, and (5) had a pre-existing diagnosis within the spectrum of “mental and behavioral disorders” (ICD-10: F00-F99). Primary diagnoses (i.e., treatment diagnoses), comorbid psychiatric and somatic diagnoses were determined by their treating clinicians (psychiatric residents, board-certified psychiatrists, psychologists, or licensed psychotherapists). At baseline (*T*_*1*_: April/May 2020), *N* = 213 patients were included, of which *N* = 159 participated in the follow-up measurement (*T*_*2*_: November/December 2020; 74.6% follow-up participation rate, please see results section for details and dropout analysis). *N* = 54 patients were lost to follow-up or refrained from participation at T_2_.

Written informed consent was obtained from all patients at *T*_*1*_. Participants were re-contacted at *T*_*2*_ according to their previous consent.

### Study design

All participants were interviewed via telephone twice in 2020 during the COVID-19 pandemic, in Lower Saxony, Germany: (1) baseline: in an early phase of the pandemic, i.e. during the first phase of maximum social restrictions (*T*_*1*_, 1st lockdown; interviews from April, 24th until May, 11th 2020), and (2) follow-up: in a later, medium-term phase of the pandemic (*T*_*2*_, 2nd lockdown; interviews from November, 27th until December, 22th 2020)–please see Supplementary Material S1 for a comparison between restrictions for both lockdowns.

Interviews were exclusively performed by qualified and specialized clinicians (psychologists, psychotherapists and psychiatrists). Both for *T*_*1*_ and *T*_*2*_, all interviewers received a rater training prior to assessment.

### Study measures: the Gottingen psychosocial Burden and Symptom Inventory (Goe-BSI)

The Goettingen psychosocial Burden and Symptom Inventory (Goe-BSI) was applied as a standardized and structured telephone interview [35]. For follow-up (*T*_*2*_), the Goe-BSI was extended from the originally 77 items (*T*_*1*_) to 84 items (*T*_*1*_*:* 77 Items) and covered the sections: (1) changes in clinical and sociodemographic data from *T*_*1*_ to *T*_*2*_, (2) COVID-19 related information (e.g., infection, testing, quarantine, risk group allocation, current symptoms), (3) psychosocial burden (primary outcome), (4) pandemic-related symptoms of an adjustment disorder (assessed by the Adjustment Disorder New Module – 20 Item version [ADNM-20] [36]), (5) general psychiatric symptoms, and (6) resilience.

Psychosocial burden was measured on a 10-point Likert scale and calculated as mean of ratings for psychosocial stress, psychiatric symptomatology and quality of life. Lower scores indicated higher psychosocial burden (inverse scale: 0: *It could not be worse*; 10: *It could not be better*). Besides self-ratings for the current state at the end of the first lockdown (April/May 2020), the first assessment (*T*_*1*_) also comprised retrospective ratings for the beginning of the pandemic with maximum lockdown restrictions (mid-March 2020) and a pre-pandemic time-point (beginning of 2020). At follow-up (*T*_*2*_, November/December 2020, i.e. 2nd lockdown) patients rated these items exclusively for their *current* state. In sum, each patient contributed four ratings to generate a course of psychosocial burden from pre-pandemic to current states in late 2020 (please see Supplementary Material S1). At *T*_*2*_, Cronbach’s alpha yielded a good internal consistency (α = 0.89) for the primary outcome of psychosocial burden, comparable to the assessments at *T*_*1*_—please see our previous publication for details about concurrent validity and reliability of the Goe-BSI [35]*.*

The ADNM-20 was used to determine psychological reactions to stressful life events—predefining the pandemic as such an event. A total of 20 items measured cognitive, behavioral and emotional reactions to the stressor (scale from 1: *never* to 4: *often*; sum score from 20 to 80 points). A cut-off of 48 points denotes individuals at high risk for an adjustment disorder [37]. The ADNM-20 was performed twice (current states at *T*_*1*_; i.e. 1st lockdown, and *T*_*2*_, i.e. 2nd lockdown, respectively).

The pandemic-related change of general psychiatric symptoms was measured at *T*_*1*_ and *T*_*2*_ with a total of 22 items. Answers ranged from 0 (*strongly disagree*) to 10 (*strongly agree*). Two resilience items used the same scale (please see Supplementary Table S2 for item formulations). Exclusively for *T*_*2*_, patients were given pre-selected 13 exemplary strategies and activities (e.g., “Housework and gardening”) and were asked if they perceived them as “particularly helpful during the Corona crisis” on a scale from 0 (*not helpful at all*) to 10 (*completely helpful*; see Supplementary Table S3).

### Statistical analyses

Data analysis was performed with IBM SPSS Statistics 26. Means (*M*), standard deviations (SD) and Pearson correlations (*r*)^1^ were computed for descriptive representation of metric variables. The primary outcome of this study (course of psychosocial burden) was analyzed via multiple general linear models (GLM) for repeated measures. Intra-individual measurements were added as four-staged within-subjects factor: each participant contributed three measurements at baseline (*T*_*1*_: *pre-*pandemic, *at the beginning* of the pandemic, *current state* during the 1st lockdown), and one measurement at follow-up (*T*_*2*_: *current state* during the 2nd lockdown). Multiple between-subjects factors (e.g., gender, ICD-10 F-axes) were subsequently added (please see results section for a detailed description of each GLM). Missing data can be derived from degrees of freedom for each model. For multiple comparisons, *p*-values were corrected within each model, using the Bonferroni method (initial significance: *p* < 0.05, two-tailed). Additionally, exploratory analyses for secondary outcomes (course of the ADNM-20 sum score, change in general psychiatric symptoms, resilience) from *T*_*1*_ to *T*_*2*_ were performed (please see results section for details).

## Results

### Baseline characteristics of the study sample

A total of *N* = 159 patients treated at the Department of Psychiatry and Psychotherapy, University Medical Center Goettingen were interviewed by telephone with the Goe-BSI both at baseline (*T*_*1*_*:* 1st lockdown, April/May 2020,) and at follow-up (*T*_*2*_: 2nd lockdown, November/December 2020,). The five most frequent primary diagnoses (according to ICD-10) were (1) F64.0 (*n* = 23, 14.5%), (2) F33.2 (*n* = 15, 9.4%), (3) F20.0 (*n* = 13, 8.2%), (4) F84.5 (*n* = 11, 6.9%), and (5) F31.1/F33.1 (each *n* = 14, 6.3%) – please see Table 1 for details and for psychotropic medication. Pooled by F-axes, frequencies of the main F-axes were as follows: (1) affective disorders (F3, *n* = 61, 38.4%), (2) disorders of adult personality and behavior (F6, *n* = 28, 17.6%), (3) neurotic, stress-related and somatoform disorders (F4, *n* = 25, 15.7%), (4) schizophrenia, schizotypal and delusional disorders (F2, *n* = 23, 14.5%), and (5) disorders of psychological development” (F8, *n* = 15, 9.4%). Patients were *M* = 41.13 years old (SD = 15.95, range 18–82 years). The relative majority was male (*n* = 72, 45.3%), followed by* n* = 64 female patients (40.3%) and *n* = 23 (14.5%) patients of non-binary gender and/or with gender identity disorder (ICD-10: F64.*). Please see Table 2 for a summary of demographic variables and outcome parameters assessed at baseline.

**Table 1:** Clinical characterization of the study sample

**(A) Main F-diagnoses (ICD-10)**		
F20.0 Paranoid schizophrenia		13 (8.2%)
F31.3 Bipolar affective disorder, manic episode		10 (6.3%)
F32.2 Severe depressive episode		8 (5.0%)
F33.1 Recurrent depressive disorder, moderate episode		10 (6.3%)
F33.2 Recurrent depressive disorder, severe episode		15 (9.4%)
F43.1 Post-traumatic stress disorder		7 (4.4%)
F64.0 Transsexualism		23 (14.5%)
F84.5 Asperger’s syndrome		11 (6.9%)
Others		62 (39.0%)

**(B) Psychotropic medication**		
Antidepressant	SSRI	43 (27.0%)
	SNRI	0 (0.0%)
	SSNRI	29 (18.2%)
	Tricyclic	6 (3.8%)
	Tetracyclic	16 (10.1%)
	Others^1^	11 (6.9%)
	Combination^2^	18 (11.3%)
	None	73 (45.9%)
Antipsychotic	Typical	2 (1.3%)
	Atypical	57 (35.8%)
	Combination	1 (0.6%)
	None	101 (63.5%)
Other	Mood stabilizer	24 (15.1%)
	Anti-dementia	1 (0.6%)
	Benzodiazepine	9 (5.7%)

Frequency (%). (A) F-diagnoses *n* ≤ 5 are summarized as “others”; (B) frequencies of psychotropic medication adds up to > 100% due to combination therapies

*SSRI* selective serotonin reuptake inhibitors, *SNRI* serotonin and norepinephrine reuptake inhibitors, *SSNRI* selective serotonin and norepinephrine reuptake inhibitors

^1^category “other antidepressants” (serotonin modulator, dual serotonergic antidepressants, MAO-inhibitor, atypical)

^2^combination of two or more antidepressants; *N* = 159 patients

**Table 2:** Demographic variables and outcome parameters at baseline

Variables measured at *T*_1_	Baseline sample: Q2/2020 (*T*_1_)	Follow-up sample: Q4/2020 (*T*_2_)		*p*^4^
	Sample *T*_1_ (*N* = 213)	Sample *T*_2_ (*N* = 159)	Dropouts *T*_2_ (*n* = 54)	
**Sociodemographic variables**				
1. Age	*M* = *42.24* ± *16.93*	*M* = 41.13 ± 15.95	*M* = 45.52 ± 19.31	0.100
2. Gender (male: female; %)	94:91 (44.1%, 42.7%)	72:64 (45.3%, 40.3%)	22:27 (40.7%, 50.0%)	0.334
3. Living space (in m^2^)	*M* = 92.47 ± 55.69	*M* = 92.47 ± 53.53	*M* = 90.57 ± 62.38	0.837
4. COVID-19 risk group (yes: no; %)	73:140 (34.3%, 65.7%)	55:104 (34.6%, 65.4%)	18:36 (33.3%, 66.7%)	0.866
**Psychosocial burden**				
5. ^1^Before the pandemic	*M* = 6.15 ± 2.03	*M* = 6.17 ± 2.01	*M* = 6.09 ± 2.08	0.812
6. ^1^Beginning of the pandemic	*M* = 5.30 ± 2.03	*M* = 5.32 ± 1.98	*M* = 5.22 ± 2.20	0.771
7. ^1^Current state (1st lockdown Q2/2021)	*M* = 5.62 ± 2.25	*M* = 5.49 ± 2.22	*M* = 5.99 ± 2.33	0.160
**ADNM-20**				
8. ADNM-20 sum score	*M* = 42.84 ± 14.07	*M* = 43.20 ± 13.88	*M* = 41.77 ± 14.74	0.524
**Resilience**				
9. ^2^ Positive changes	*M* = 4.41 ± 3.59	*M* = 4.47 ± 3.56	*M* = 4.24 ± 3.72	0.692
10. ^2^Opportunities	*M* = 3.14 ± 3.50	*M* = 3.21 ± 3.50	*M* = 2.93 ± 3.52	0.609
**F-Axes (ICD-10)**^3^				
11. Multiple F-diagnoses (yes: no; %)	119:94 (55.9%, 44.1%)	86:73 (54.1%, 45.9%)	33:21 (61.1%, 38.9%)	0.369
12. F2 Schizophrenia, schizotypal and delusional disorders	*n* = 31 (14.6%)	*n* = 23 (14.5%)	*n* = 8 (14.8%)	
13. F3 Affective disorders	*n* = 78 (36.6%)	*n* = 61 (38.4%)	*n* = 17 (31.5%)	
14. F4 Neurotic, stress-related and somatoform disorders	*n* = 35 (16.4%)	*n* = 25 (15.7%)	*n* = 10 (18.5%)	0.913
15. F6 Disorders of adult personality and behavior	*n* = 35 (16.4%)	*n* = 28 (17.6%)	*n* = 7 (13.0%)	
16. F8 Disorders of psychological development	*n* = 20 (9.4%)	*n* = 15 (9.4%)	*n* = 5 (9.3%)	

Data presented as means (*M*), standard deviations (± *SD*), and frequencies. Captions: *Gender* (binary: male = 1, female = 2); *risk group* for a severe course of COVID-19 (yes = 1, no = 2); *ADNM-20* sum score (20 to 80 points)

^1^Psychosocial burden: items rated from 0 to 10, low scores denote high psychosocial burden

^2^Resilience: items rated from 0 to 10

^3^Allocation of all F-diagnoses to the corresponding F-axes (F-axes F0, F1, F5, and F9 were excluded from this analysis due to small sample size)

^4^Uncorrected *p*-values for metric variables (*t* tests), and binary variables (2 × 2/2 × 5 χ^2^-tests) between sample *T*_*2*_ and dropouts *T*_*2*_

A total of *n* = 55 patients (34.6%) belonged to a risk group for a severe course of a SARS-CoV-2 infection (self-report). Until follow-up, *n* = 51 (32.1%) had been tested for Covid-19 (*n* = 1 positive), *n* = 8 (5.0%) had temporary contact to a positively tested person, *n* = 5 (3.1%) had been quarantined, and *n* = 22 (13.8%) had temporarily resided in a Corona risk area. The majority of patients declared that daily living conditions during the pandemic were stable (family status: 93.1%, living conditions: 85.5%, occupational situation: 78.0%, financial situation: 80.5%, childcare: 89.3%). According to the patients’ self-report, the majority of psychiatric diagnoses remained unchanged (*n* = 142, 89.3%). Of those reporting changes, *n* = 7 (41.2%) specified an improvement and *n* = 10 (58.8%) a worsening of their mental health condition.

From *T*_*1*_ to *T*_*2*_, a total of *n* = 54 patients (25.4%) dropped out of the study. The majority of these were lost to follow-up (*n* = 34, 63.0%), *n* = 16 (29.6%) withdrew consent at *T*_*2*_, and *n* = 4 (7.4%) had died between *T*_*1*_ and *T*_*2*_*.* A dropout analysis was performed on sociodemographic variables and primary/secondary outcomes of this study, revealing no significant differences between the follow-up- and dropout-sample for all parameters (*p*-values between 0.100 and 0.913, *ns*, see Table 2 for details).

### Medium-term and peri-lockdown course of psychosocial burden

#### Total sample

**Fig. 1:**
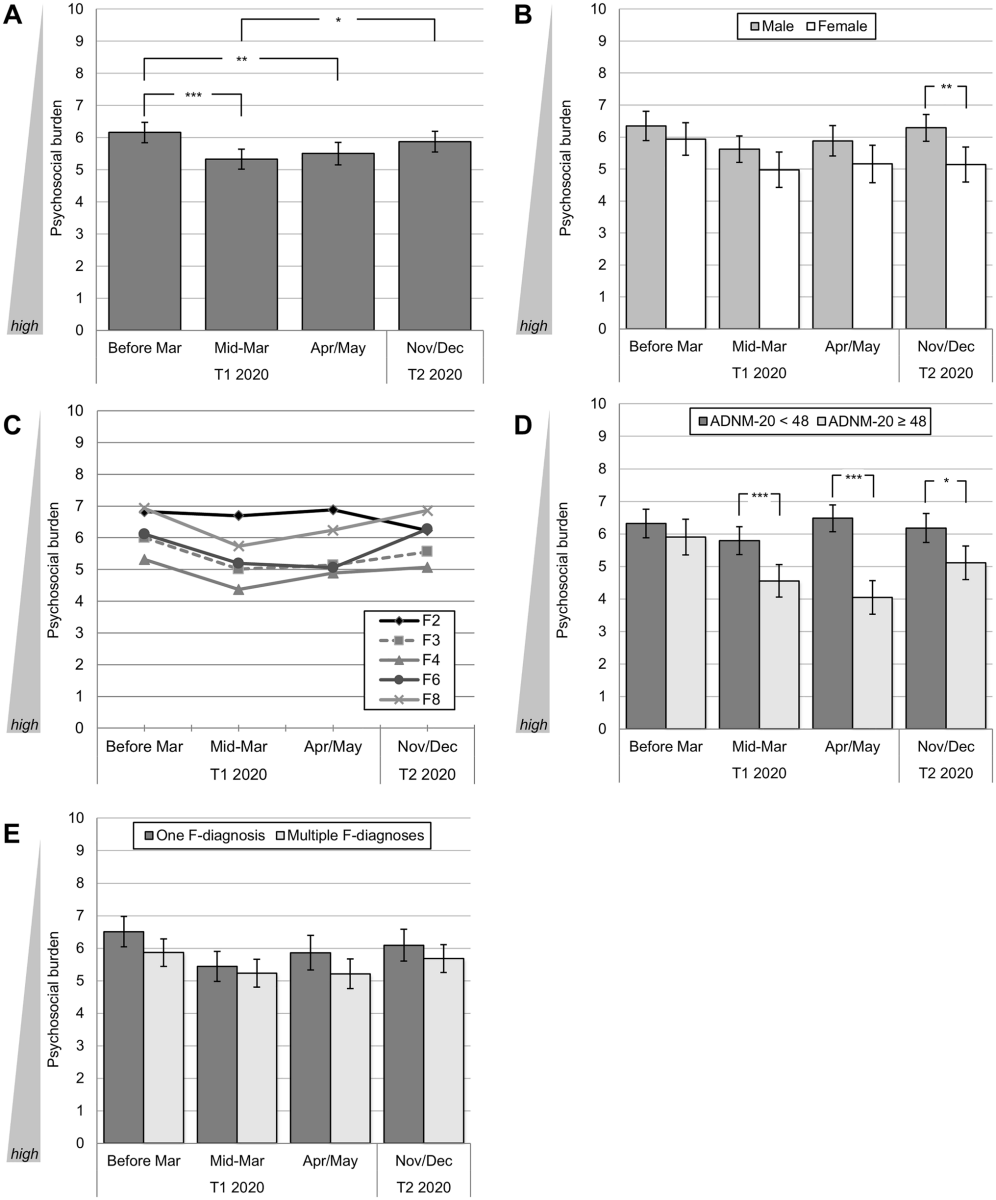
Medium-term and peri-pandemic course of psychosocial burden in patients with pre-existing mental disorders. **A** Course of the total sample (*N* = 156); differentiated by **B** gender (binary), *N* = 133; **C** ICD-10 F-axes (*F2* to *F8*), *N* = 132; **D** ADNM-20 cut-off value indicating a high risk for adjustment disorder, *N* = 149; **E** psychiatric comorbidities: one vs. multiple F-diagnoses, *N* = 156.* p < 0.05, ** *p* < 0.01, *** *p* < 0.001. Mean values with 95%-CIs (**A, B, D, E**) and Bonferroni-corrected pairwise comparisons (**A, B, D, E**). Psychosocial burden is presented as mean of ratings on the 10-point Likert scales for psychosocial stress, psychiatric symptomatology, and quality of life. Ratings comprised pre-pandemic estimates (early 2020, retrospective rating), ratings very early during the pandemic/first lockdown (mid-March 2020, retrospective rating), at the end of the first lockdown (April/May 2020), and for the *current* state during the second lockdown (November/December 2020)

The GLM revealed a significant variation of psychosocial burden over time (*F*(3, 465) = 9.26. *p* < 0.001, partial η^2^ = 0.06; see Fig. 1A): Psychosocial burden significantly increased from time-points *before* the pandemic (*M* = 6.16, SD = 2.02) to its *beginning* (mid-March 2020, *M* = 5.33, SD = 1.99; *p* < 0.001), and decreased at the end of the first lockdown (April/May 2021, *T*_*1*_: *M* = 5.51, SD = 2.23), as reported by Belz et al. [35]. This relief was then carried over to the follow-up measurement (2nd lockdown November/December 2020, *T*_*2*_: *M* = 5.87, SD = 2.06): (1) Patients regained levels of psychosocial burden at follow-up in late 2020 comparable to those at a pre-pandemic time-point (*p* = 0.639), and (2) psychosocial burden was significantly lower at follow-up compared to elevated levels at the *beginning* of the pandemic (*p* = 0.016; see Fig. 1A).

#### Gender differences

A two-staged between-subjects factor (male vs. female^2^) was added to the GLM to analyze possible gender effects. In line with Belz et al. [35], results showed that, in general, female patients experienced higher psychosocial burden than male patients (between-subjects effect: *F*(3, 393) = 7.20, *p* < 0.001, partial η^2^ = 0.05; see Fig. 1B), but the course of psychosocial burden did not differ between both genders (interaction effect: *F*(3, 393) = 1.34, *ns*). However, pairwise comparisons yielded a significant difference between both genders during the second lockdown (*T*_*2*_; *M*_Diff_ = 1.14, *p* = 0.005): At this time-point, male patients had approximately reached their initial (lower) level of psychosocial burden assessed at *T*_*1*_ (*M*_Diff_ = 0.06), whereas female patients showed a considerably higher burden in relation to their baseline level (*M*_*Diff*_ = − 0.79).

#### Differences between ICD-10 F-axes

A five-staged between-subjects factor for the different ICD-10 F-axes (F2, F3, F4, F6, and F8^3^) was added to the GLM. Similarly to our previous findings [35], the general level of psychosocial burden differed significantly between F-axes (*F*(4, 144) = 5.13, *p* < 0.001, partial η^2^ = 0.13, see Fig. 1C). The highest level for all time-points was found for the F4-axis (*M* = 4.91), the lowest level for the F2-axis (*M* = 6.66). Significance was only reached for the pairwise comparisons between F2- vs. F3-/F4-axes (*p* = 0.014, *p* < 0.001), and, additionally, for those between F4- vs. F8-axes (*p* = 0.035; all other pairwise comparisons *ns*).

However, overall courses of psychosocial burden between F-axes were found to be non-diverging over time (interaction effect: *F*(12, 432) = 1.22, *ns*). As displayed in Fig. 1C, all F-axes showed a numerical reduction of psychosocial burden from the first to the second lockdown. As only exception, psychosocial burden levels of the F2-axis increased (*M*_Diff_ = − 0.65).

#### Risk groups by ADNM-20

The sample was divided into groups at high (*n* = 50) vs. low risk (*n* = 82) for adjustment disorder by ADNM-20 values assessed at April/May 2020 (cut-off ≥ 48 [37]). A two-staged between-subjects factor (high risk vs. low risk) was then added to a GLM, together with gender (binary) as covariate, due to a high correlation of binary gender with ADNM-20 baseline scores (*r* = 0.308, *p* < 0.001). In line with Belz et al. [35], a significant between-subjects effect for the high- vs. low risk subgroup was found (GLM: *F*(3, 387) = 3.72, *p* = 0.012, partial η^2^ = 0.03), together with a significant interaction effect (GLM: *F*(3, 387) = 10.76, *p* < 0.001, partial η^2^ = 0.08). As illustrated in Fig. 1D, patients at high risk for an adjustment disorder experienced a continuous increase of psychosocial burden from pre-pandemic time-points (*M* = 5.90, SD = 1.97) over the early weeks of the first lockdown (*M* = 4.55, *SD* = 1.81) until the end of the first lockdown (*M* = 4.05, SD = 1.87). Both for the early phase in mid-March 2020 (*M*_Diff_ = 1.24,* p* < 0.001) and at the end of the first lockdown in April/May 2020 (*M*_Diff_ = 2.43, *p* < 0.001), their burden was significantly higher compared to patients at low risk for an adjustment disorder. At follow-up during the second lockdown (*T*_*2*_), this difference remained significant (*M*_Diff_ = 1.07, *p* = 0.013) although cut by more than half compared to the first lockdown.

#### Risk groups by psychiatric comorbidities

Patients were assigned to the two subgroups (1) “one F-diagnosis” (*n* = 73) and (2) “two or more F-diagnoses (*n* = 86) at baseline (*T*_*1*_). A GLM indicated a trend difference for psychosocial burden (between-subjects effect: *F*(1, 154) = 3.48, *p* = 0.064, partial η^2^ = 0.02, all other effects *ns*). As displayed in Fig. 1E, patients with multiple F-diagnoses showed a numerically higher level for all measurements, although all corrected pairwise comparisons missed statistical significance.

### Descriptive results and exploratory analyses of secondary outcomes

#### Course of ADNM-20 sum scores from the first to the second lockdown

**Fig. 2:**
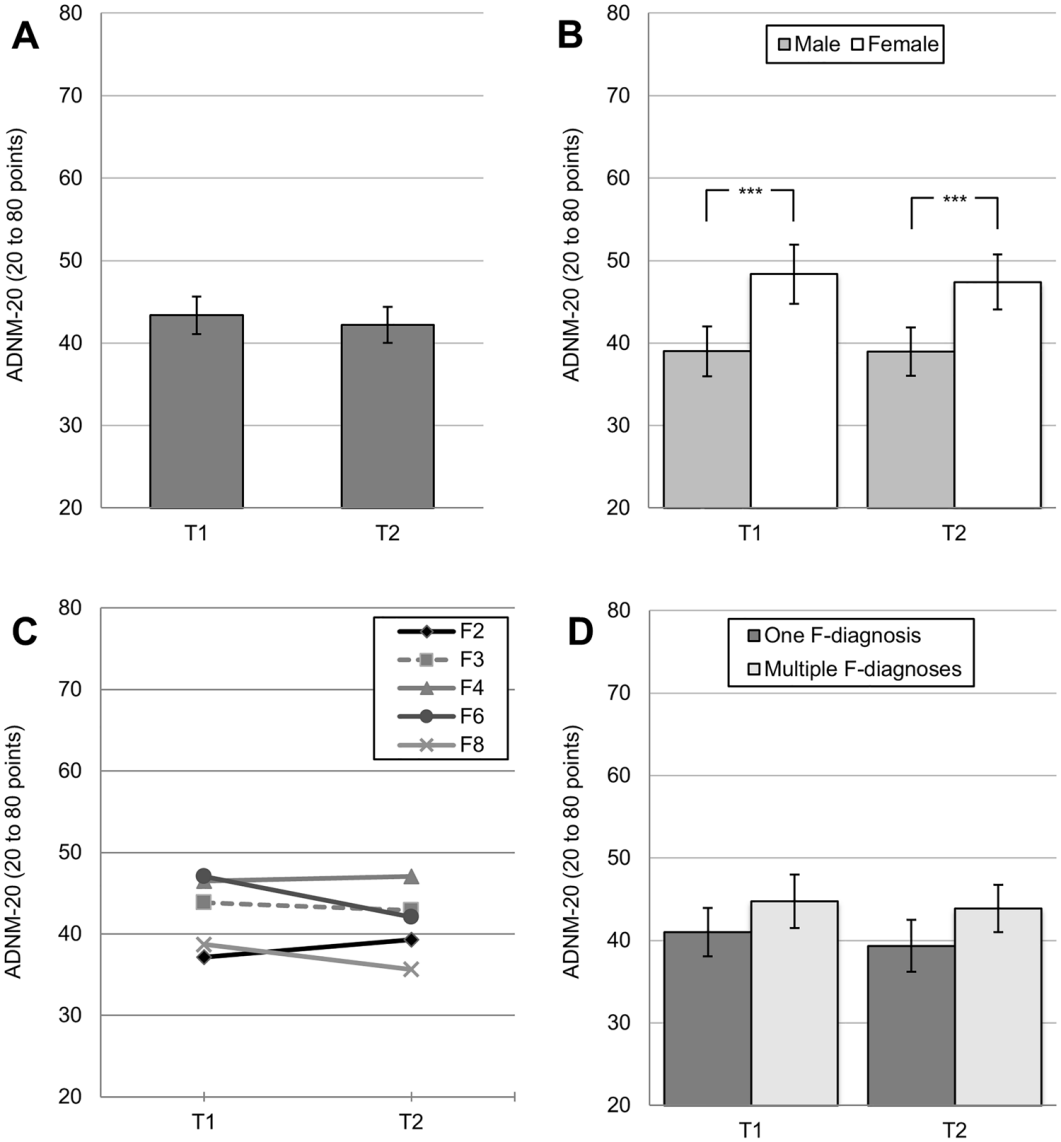
Medium-term and peri-pandemic course of symptom levels of adjustment disorder measured by the ADNM-20 in patients with pre-existing mental disorders, for *T*_*1*_ (1st lockdown, April/May 2020) and *T*_*2*_ (2nd lockdown, November/December 2020), **A** for the total sample (*N* = 152); differentiated by **B** gender (binary), *N* = 130; **C** ICD-10 F-axes (*F2* to *F8*), *N* = 145; **D** psychiatric comorbidities: one vs. multiple F-diagnoses, *N* = 152. * p < 0.05, ** *p* < 0.01, *** *p* < 0.001. Mean values with 95% CIs and Bonferroni-corrected pairwise comparisons for the ADNM-20 sum score (range 20 to 80 points)

Please see Table 3 for correlations between secondary outcomes at follow-up (*T*_*2*_) and baseline sociodemographic variables. For the entire sample, the ADNM-20 sum score numerically decreased by *M*_Diff_= 1.28 points but did not vary significantly from the end of first lockdown (*T*_*1*_; *M* = 43.01, SD = 13.92) to the follow-up during the second lockdown (*T*_*2*_: *M* = 41.74, SD = 13.46; GLM: *F*(1, 151) = 1.88, *ns*; see Fig. 2A). Due to a significant correlation of binary gender and ADNM-20 scores (*T*_*1*_, *r* = 0.322, *p* < 0.001, *T*_*2*_: *r* = 0.311, *p* < 0.001), a two-staged between-subjects factor [male vs. female (see footnote 2)] was added to the GLM, and revealed general differences between genders (GLM: *F*(1, 128) = 17.38, *p* < 0.001, partial η^2^ = 0.12): Both, for *T*_*1*_ and *T*_*2*_, female patients showed significantly higher ADNM-20 sum scores (all *p*-values < 0.001; see Fig. 2B). Analyses did not reveal a significant interaction effect, indicating a homogenous course of ADNM-20 sum scores for both genders (GLM: *F*(1, 128) = 0.23, *ns*). An additional GLM was created to differentiate between F-axes [F2, F3, F4, F6, F8 (see footnote 3)], but no significant effects (within-subjects, between-subjects, interaction) were found (all F-values *ns*; see Fig. 2C). Again, the two subgroups (1) “one F-diagnosis” vs. (2) “two or more F-diagnoses” were added as between-subjects factor to an additional GLM. Results showed a significant between-subjects effect (*F*(1, 150) = 4.29, *p* = 0.040, partial η^2^ = 0.03; pairwise comparisons *ns*). As shown in Fig. 2D, patients with multiple F-diagnoses showed a generally higher level of adjustment disorder symptoms.

**Table 3:** Correlations between sociodemographic variables and secondary endpoints

Variable	1	2	3	4	5	6	7	8	9	10	11	12
**Sociodemographic variables**												
1. Multiple F-diagnoses	–											
2. Age (in years)	− 0.003	–										
3. Gender	− 0.007	0.200^*^	–									
4. Living space (in m^2^)	− 0.162^*^	0.101	0.036	–								
5. COVID-19 risk group	− 0.033	− 0.492^**^	− 0.198^*^	− 0.010	–							
**ADNM-20** at *T*_*2*_												
6. ADNM-20 sum score	0.175^*^	0.058	0.311^**^	0.051	− 0.113	–						
**Most pronounced psychiatric symptoms** at *T*_*2*_												
7. ^1^Vigilance	0.131	0.037	0.092	0.058	− 0.173^*^	0.552^**^	–					
8. ^1^Self-observing of disease symptoms	0.069	0.039	0.151	0.145	− 0.030	0.488^**^	0.456^**^	–				
9. ^1^Observing disease symptoms of others	0.109	− 0.049	0.096	0.219^**^	0.002	0.420^**^	0.478^**^	0.662^**^	–			
10. ^1^Being physically inactive	0.112	0.041	0.270^**^	− 0.054	− 0.096	0.503^**^	0.366^**^	0.301^**^	0.243^**^	–		
11. ^1^Internet/media use	0.115	− 0.272^**^	0.098	− 0.161	− 0.011	0.326^**^	0.309^**^	0.291^**^	0.272^**^	0.304^**^	–	
**Resilience** at *T*_*2*_												
12. ^2^Positive changes	− 0.078	− 0.271^**^	− 0.106	− 0.063	0.128	− 0.180^*^	− 0.069	0.079	0.118	− 0.387^**^	0.154	–
13. ^2^Opportunities	− 0.033	− 0.199^*^	− 0.098	0.059	0.116	− 0.210^**^	− 0.081	0.105	0.086	− 0.367^**^	0.029	0.631^**^

Correlations: *p < 0.05. **p < 0.01. Captions: *Multiple F-diagnoses* at baseline (0 = one F-diagnosis, 1 = multiple F-diagnoses); *Gender* (male = 1, female = 2); *risk group* for a severe course of COVID-19 (yes = 1, no = 2); variables measured at *T*_*2*_ (follow-up: 2nd lockdown Q4/2020): *ADNM-20* sum score (20 to 80 points)

^1^most pronounced psychiatric symptoms: items rated from 0 to 10

^2^resilience: items rated from 0 to 10. (*N* = 136; *df* = 134 to *N* = 159; *df* = 157)

#### Course of general psychiatric symptoms and resilience

A slight shift of the five most pronounced psychiatric symptoms from the first (*T*_*1*_) to the second lockdown (*T*_*2*_) was observed: At *T*_*1*_ vigilance, media use, observing disease symptoms in others, poor drive, and self-observation for disease symptoms were among the most intense symptoms, whereas at *T*_*2*_ vigilance, self-observation, observing others, being physically inactive, and media use were most pronounced (Supplementary Table S2). These five symptoms at *T*_*2*_ were positively correlated (all *p* < 0.01). Please see Table 3 for all correlations.

Differences for 22 pre-defined psychiatric symptoms were analyzed between baseline (*T*_*1*_*:* April/March 2020, end of 1st lockdown) and follow-up (*T*_*2*_: November/December 2020, 2nd lockdown)—please see Supplementary Table S2 for all pairwise comparisons. Significant changes were restricted to four symptoms: During follow-up, patients (1) payed more attention to symptoms of illness in others (*M*_Diff_ = 0.79, *p* = 0.003), (2) screened themselves more often for signs of illness (*M*_Diff_  = 0.96, *p* < 0.001), (3) reduced initial changes in their eating behavior (*M*_Diff_ = − 0.83, *p* = 0.005), and (4) showed more self-injurious behavior (*M*_Diff_ = 0.23, *p* = 0.049).

Regarding resilience, the items referring to positive changes and opportunities during the pandemic were rated significantly lower as the pandemic continued: patients reported less “*things have changed in a positive way during the pandemic*” from baseline (*T*_*1*_) to follow-up measurement (*T*_*2*_; *M*_Diff_ = − 0.64, *p* = 0.044), along with the perception of significantly fewer “*opportunities*” at follow-up (*T*_*2*_; *M*_Diff_ = − 0.62, *p* = 0.037; see Supplementary Table S2). Older age was negatively correlated with both resilience items (*p* < 0.05 and < 0.01), as was the ADNM-20 score (*p* < 0.05 and < 0.01), and the psychiatric symptom “*poor drive*” (all *p* < 0.01). Please see Table 3 for details. At follow-up, patients had to specify strategies and activities being “*particularly helpful during the Corona crisis*”—please see Supplementary Table S3 for an English translation and descriptive data. The five items which were rated as being most helpful were: (1) contact with friends/family (*M* = 6.65), (2) hobbies (*M* = 5.87), (3) contact with psychiatrist/psychotherapist (*M* = 5.78), (4) fewer appointments (*M* = 4.79), and (5) being alone (*M* = 4.54).

## Discussion

The continuing pandemic and its societal restrictions during recurrent lockdown periods as significant life-changing stressors put strong demands on an individual’s adaptability. In this light, careful long-term observation of groups at particular risk for worsening of their mental health status or for emergence of new symptoms—such as patients with pre-existing mental disorders—is crucial. Longitudinal, peri-pandemic data on mental health is steadily accumulating but so far largely limited to time frames up to summer 2020 when governmental measures were eased after the first lockdown in countries worldwide. This longitudinal study investigated the medium-term und peri-lockdown course of psychosocial burden, psychiatric symptoms and resilience from pre-pandemic estimates to states during the second lockdown in November/December 2020. Most importantly, after an initial rise, psychosocial burden returned to pre-pandemic levels suggesting an adaptive stress response, and was paralleled by a numerical decrease in adjustment disorder symptoms from the first to the second lockdown. However, at the same time, diminishing resilience requires ongoing and careful attention on individuals’ mental health status.

### Follow-up of psychosocial burden and adjustment disorder symptomatology

The distinctive course of psychosocial burden over time reported here may at least partially be interpreted as a normal stress response. Recent studies in the general population also demonstrated similar patterns of psychological reactions during the early phase of the pandemic [38–41] until summer 2020 [34, 42]. On the other hand, this reaction pattern seems to mimic symptoms of adjustment disorder, a syndrome implying a strong response to acute or persisting external stressors that manifests in anxiety, mild depression and stress symptoms. In most cases, these symptoms are transient and attenuate as a result of an adaption process. Increased levels of anxiety, depression and (post-traumatic) stress-related symptoms in an early phase of the pandemic as reported by others (e.g. [20, 21, 43–47]) could possibly be explained by such an adaptive process to this external stressor. Alternatively, these changes might also be interpreted as exacerbations of pre-existing mental disorders since concurrent depression and anxiety disorders preclude the diagnosis of an adjustment disorder [48]. Accordingly, the ADNM-20 was used here as a screening measure, not as diagnostic instrument.

For a general integration of our main result—showing a return of psychosocial burden to pre-pandemic levels—into the current empirical context, study types and temporal dynamics of the pandemic in relation to assessment periods have to be considered. A majority of cross-sectional studies were conducted in the early phase of the pandemic when the first lockdown had been enacted (mid-March to mid-May 2020) and reported poor mental health outcomes (e.g. in anxiety, depression, stress, quality of life measures) in patients with pre-existing mental disorders [19, 43–45, 49]. In two cross-sectional studies, these patients even showed high symptom levels post-lockdown (mid-May to September 2020 [46, 50]). Few longitudinal studies point in the same direction and present previous mental disorders as a risk factor for worse mental health outcomes [47], their deterioration over time [20] or accelerated referral rates to secondary health services [51]. Mixed results were demonstrated by Robillard et al. [21] post-lockdown (late June 2020) with less worsening of anxiety but higher worsening of depression symptoms in association with longer time elapsed since the beginning of the pandemic. Echoing our findings, several longitudinal surveys during the first weeks of the pandemic (late March to mid-May 2020), found only a modest increase in symptomatology [23], no changes [24, 25, 34] or even a decrease over time [39] although patients with premorbid mental disorders displayed higher symptom levels in most cases compared to controls. Even for longer time frames post-lockdown (June to late October 2020), there is first evidence for stable [26, 27] or attenuating mental health states [38, 42, 52] that seems to extend to the second lockdown in late 2020 as suggested by our results. Thus, high symptom levels or a worsening of mental health outcomes during the very early phase of the pandemic are not necessarily in conflict with the present medium-term and peri-lockdown data if subsequent adaptation processes and longer observation periods are considered.

### De novo onset of mental disorders and changes in pre-existing psychopathology

With the rise of the pandemic, considerable concern has been repeatedly raised about the de novo onset of psychiatric symptoms and disorders in individuals with and without pre-existing mental disorders [1, 9, 10]. It seems plausible to deduce that resilience capacities will be ultimately depleted if mental health is strongly and persistently challenged. In this study, the primary purpose was to closely monitor the course of psychosocial burden in patients with pre-existing mental disorders and not to study their de novo onset. Nevertheless, we also asked for self-report if the patients’ mental health condition worsened or if new psychiatric disorders were diagnosed. Overall, no notable increase was found and psychosocial burden returned to pre-pandemic levels. Additionally, we only noticed a modest shift of most pronounced psychiatric symptoms mainly attributable to a higher awareness of somatic disease symptoms, drive/physical activity and media use. Importantly, an increase in self-injurious behavior should deserve high attention in the clinical setting. However, an increased prevalence of psychiatric disorders in formerly mentally healthy subjects cannot be excluded by this study as it may be obscured by barriers in accessing appropriate treatment during lockdown periods [53] or may occur later on. Also, patients with premorbid mental disorders may experience a worsening of symptoms and psychosocial burden at time-points later than those covered here.

### Risk and resilience factors for mental health outcomes

Female gender has been repeatedly identified as a risk factor for worse mental health outcomes [21, 38, 39, 47] and matches our own results that demonstrate an unfavorable course of psychosocial burden and higher levels of adjustment disorder symptoms in female compared to male individuals. The risk factor of a high initial adjustment disorder symptomatology reported previously [35] attenuated over time. So far it remains unclear if this trend will continue with the ongoing pandemic or may be overcome by decreasing resilience.

Recent studies found that psychopathological syndromes are differentially affected by pandemic-related stressors and that particular disorders show associations with even poorer outcomes over the course of the pandemic than others. Descriptive data of our sample also point to a particularly high level of psychosocial burden in patients with disorders of the F4-axis, and such are in principal compatible with findings reported for anxiety disorders [52, 54].

Patients with psychiatric comorbidities (i.e., multiple F-diagnoses) experienced a trend towards a higher impact on psychosocial burden and a significant increase in adjustment disorder symptoms over time. Tsamakis et al. [55] reported that patients with severe premorbid mental disorders (severe depression, schizophrenia) showed a considerable resilience without worsening of their pre-pandemic condition but these findings refer to earlier phases of the pandemic. It is, thus, possible that also severely affected patients may preserve their mental health status in the short-term until resilience capacities are finally worn out. Disease severity—if operationalized by comorbidities—may, therefore, be regarded as a risk factor for poor clinical outcomes. However, it currently remains an open question whether the total of individuals with pre-existing mental disorders or diagnostic subgroups thereof are at risk for worse mental health outcomes.

Additionally, we assessed resilience factors which may support coping with pandemic-related stressors more extensively at follow-up. Besides social contacts (friends/family) and hobbies, contact with professional therapists was among the most helpful strategies. Thus, maintaining treatment continuity in addition to pandemic- and risk factor-specific treatment interventions could be most important. Accordingly, Methfessel et al. reported a severe clinical deterioration related to electroconvulsive therapy discontinuation or reduction during the pandemic in severely ill patients [56]. In parallel to positive longitudinal outcomes on psychosocial burden, we observed a decrease in general resilience ratings from the first to the second lockdown. From our data, considerable resilience still seems available to sufficiently stabilize psychosocial burden until the end of 2020. However, further prospective follow-ups are needed to simultaneously monitor the balance of both factors.

## Strengths and limitations

Strengths and limitations are detailed in Belz et al. [35]. In summary, diagnoses validated by treating clinicians instead of self-reported diagnoses rank among the major strengths of our approach. Furthermore, the Goe-BSI has repeatedly proven to be a valid and reliable psychometric instrument sensitive to detect changes. Longitudinal data—as presented here—allow to track changes in psychosocial burden from pre-pandemic ratings over two lockdown periods. Narrow time-windows of data acquisition additionally enabled observations under constant lockdown restrictions. Dropout analyses revealed that patients lost to follow-up did not differ from patients included in the present analyses in terms of sociodemographic variables as well as primary and secondary outcomes for the first assessment in April/May 2020.

Data presented here exclusively focused on the peri-pandemic course of a clinical sample (patients with pre-existing mental disorders). This study did, thus, not address the development of mental health problems in healthy individuals due to reasons of feasibility and methodological considerations (timely recruitment of a large sample under constant lockdown conditions). In this context, it remains a matter of discussion if healthy individuals potentially experience even more pandemic-related stressors (e.g. health-related worries, social, financial and occupational adversities). In the absence of own data on healthy individuals for comparison, data on the general (healthy) population provided or summarized by others was used as a reference. Multiple studies with healthy participants support the observed course here, indicative of a normal stress response with an initially increased mental load followed by a relief [34, 38–42]. Also, partitioning patients by ICD-10 F-axes allowed comparisons of clinical subgroups who exhibited similar courses of mental health outcomes as reported above. Further limitations mainly refer to the composition of our outpatient convenience sample and the distribution of mental disorders within ICD-10 F-axes. A disproportionate inclusion of patients with autism spectrum disorder (F8) and gender identity disorder (ICD-10: F64. *) can be explained by specializations of our department. However, this inclusion bias has only minor effects on our results, since a similar pattern of the primary outcome “*psychosocial burden* “ was found across all major ICD-10 F-axes (see above). Furthermore, causal inferences on pandemic-related changes have to be drawn cautiously. The Goe-BSI has been primarily developed to comprehensively assess the pandemic-related course of psychosocial burden, psychiatric symptoms and resilience, trying to balance scientific value, time and psychological load on patients. Thus, this survey does not comprise the full range of patients’ experiences. Since for the tailoring of risk group-adapted mental health interventions against unfavorable pandemic-related outcomes, it is of high relevance to investigate which factors may cause worsening or stabilization of mental health status, we enclosed additional resilience items at follow-up.

## Conclusion

A return of psychosocial burden to pre-pandemic levels in patients with pre-existing mental disorders during a time span covering more than nine months and two lockdown periods of the COVID-19 pandemic seems promising but does not necessarily preclude a later worsening or de novo onset of mental disorders in such patients. In face of the here-observed decreasing resilience, a turning-point at which coping capacities are depleted and result in clinical deterioration has to be carefully monitored longitudinally. Ideally, this turning point has to be proactively counteracted as part of a preventive endeavor. Particular efforts suitable to overcome this anticipated development, e.g., with the help of digital solutions, have to be made, especially if barriers in accessing health care services for patients with mental disorders are considered.

## Supplementary Information

Below is the link to the electronic supplementary material.

Supplementary file1 (DOCX 77 KB)

Supplementary file2 (DOCX 23 KB)

Supplementary file3 (DOCX 18 KB)

## Data Availability

The data that support the findings of this study are available on reasonable request from the corresponding author. The data are not publicly available due to privacy or ethical restrictions.

## Footnotes

1 The mean square contingency coefficient (phi coefficient) was used to calculate correlations between binary variables (e.g., *binary gender* and *risk group*). It is reported in Table 3 along with Pearson correlation coefficients for the remaining variables.

2 Explanation: Exclusion of patients with a Gender Identity Disorder (F64.0) due to confounding factors prohibiting valid assignment to biological/binary gender (e.g., hormone therapy, self-concept).

3 F-axes F0, F1, F5, and F9 had to be excluded from this analysis due to small sample sizes (*n* ≤ 3).
