# Supplementary material for: Medium-term and peri-lockdown course of psychosocial burden during the ongoing COVID-19 pandemic: a longitudinal study on patients with pre-existing mental disorders
Source: Eur Arch Psychiatry Clin Neurosci. 2021 Nov 25;272(5):757–71. doi: 10.1007/s00406-021-01351-y (PMC8614217; doi:10.1007/s00406-021-01351-y)
Supplement: Supplementary file 1 — Supplementary file1 (DOCX 77 KB) [file 406_2021_1351_MOESM1_ESM.docx]

**Supplementary Material S1**

**Title:** Medium-term and peri-lockdown course of psychosocial burden during the ongoing Covid-19 pandemic: A longitudinal study on patients with pre-existing mental disorders

Claudia Bartels PhD^1^, Philipp Hessmann MD, MPH^1^, Ulrike Schmidt MD^1,2,3^, Jonathan Vogelgsang MD^1,4^, Mirjana Ruhleder PhD^1^, Alexander Kratzenberg MSc^1^, Marit Treptow MSc^1^, Thorgund Reh-Bergen MSc^1^, Mona Abdel-Hamid PhD^1,5^, Luisa Heß MSc^1^, Miriam Meiser MD^1^, Jörg Signerski-Krieger MD^1^, Katrin Radenbach MD^1^, Sarah Trost MD^1,6^, Björn H. Schott MD, PhD^1,7,8^, Jens Wiltfang MD^1,7,9^, Claus Wolff-Menzler MD, MA^1^*^&^*, Michael Belz PhD^1^*^&^***^*^**

^1^Department of Psychiatry and Psychotherapy, University Medical Center Goettingen, Germany

^2^Department of Psychiatry and Psychotherapy, University Hospital Bonn, Germany

^3^Maastricht University Medical Center, School for Mental Health and Neuroscience, Department of Psychiatry and Neuropsychology, Maastricht, The Netherlands

^4^McLean Hospital, Harvard Medical School, Translational Neuroscience Laboratory, Belmont, MA, USA

^5^Department of Psychiatry and Psychotherapy, University of Duisburg-Essen, LVR-Hospital Essen, Germany

^6^Geriatric Psychiatry, University Department of Geriatric Medicine FELIX PLATTER, Basel, Switzerland

^7^German Center for Neurodegenerative Diseases (DZNE), Goettingen, Germany

^8^Leibniz Institute for Neurobiology, Magdeburg, Germany

^9^Neurosciences and Signaling Group, Institute of Biomedicine (iBiMED), Department of Medical Sciences, University of Aveiro, Aveiro, Portugal

*^&^both authors contributed equally to the work as senior authors.*

***Corresponding author:** Claudia Bartels, Department of Psychiatry and Psychotherapy, University Medical Center Goettingen, von-Siebold-Str. 5, D-37075 Goettingen, Germany, [claudia.bartels@med.uni-goettingen.de](mailto:claudia.bartels@med.uni-goettingen.de), +49 551 3914397

**Supplementary Material S1a** Study assessment time-points in relation to the Covid-19 pandemic (Covid-19 infection rates, 1^st^ and 2^nd^ lockdown in Germany 2020)

COVID-19 cases in Germany (2020 to 2021)


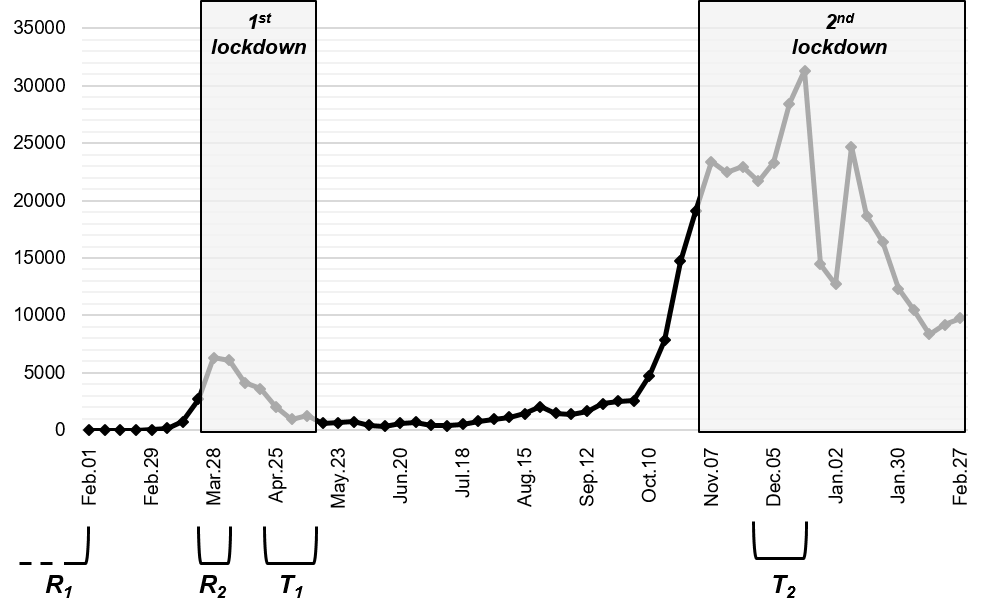


*Notes*: R_1_: retrospective rating (pre-pandemic), R_2_: retrospective rating (very early phase of the pandemic, beginning of 1^st^ lockdown, mid-March 2020, T_1_: first assessment time point (early phase of the pandemic, end of 1^st^ lockdown, April/May 2020, T_2_: second assessment time point (medium-term phase of the pandemic, 2^nd^ lockdown, November/December 2020)

**Supplementary Material S1b** Comparison of pandemic related restrictions during 1^st^ and 2^nd^ lockdown

| **1^st^ Lockdown**  March 23^rd^ until May 10^th^ ^1^ | **2^nd^ Lockdown**  From November 2^nd^ ^2^ | **2^nd^ Lockdown (tightened)**  From December 16^th^ ^3^ | |
| --- | --- | --- | --- |
| **Public sector** | | |  |
| 1.5m distance | 1.5m distance | 1.5m distance | |
| Only everyday commute, purchases for everyday needs, doctor appointments, and similar acts allowed | | |  |
| Museums, theaters etc. closed | Museums, theaters etc. closed | Museums, theaters etc. closed | |
| Schools and children day-care centers closed | Schools and (limited) children day-care open | Limited children day-care, home teaching | |
| Shops closed except for supermarkets, drug stores, filling stations | Shops opened under strict hygienic restrictions | Shops closed except for supermarkets, drug stores, filling stations | |
| Restaurants, hotels, service providers (e.g., hairdresser) closed | Restaurants closed, hotels closed (for tourists), service providers (e.g. hairdresser) open under strict hygienic restrictions | Restaurants, hotels, service providers (e.g., hairdresser) closed | |
| Sports complexes closed, sports activities prohibited (only individual outdoor sports) | Sports complexes closed, sports activities generally limited | | |
| **Individual contacts** | | |  |
| Limited to own household + 1 person of a different household | Limited to own household + multiple persons of a different household (max. 10 persons) | Limited to own household + multiple persons of a different household (max. 5 persons) | |

*Notes.* Contact between persons besides persons of the same household had to be reduced to a “minimum” in general. This was not specified due different regulations in the federal states of Germany and multiple changes in this period.

^1^<https://www.niedersachsen.de/download/153376/Allgemeinverfuegung_des_Niedersaechsischen_Gesundheitsministeriums_zur_Beschraenkung_von_Sozialen_Kontakten_vom_22.03.2020.pdf>

^2^https://www.stk.niedersachsen.de/startseite/presseinformationen/neue-corona-verordnung-zur-zweiten-welle-194059.html

^3^https://www.stk.niedersachsen.de/startseite/presseinformationen/anderungen-in-der-corona-verordnung-ab-dem-16-dezember-2020-195544.html

^4^Department of Psychiatry and Psychotherapy, University Medical Center Goettingen, Germany
