# Supplementary material for: Medium-term and peri-lockdown course of psychosocial burden during the ongoing COVID-19 pandemic: a longitudinal study on patients with pre-existing mental disorders
Source: Eur Arch Psychiatry Clin Neurosci. 2021 Nov 25;272(5):757–71. doi: 10.1007/s00406-021-01351-y (PMC8614217; doi:10.1007/s00406-021-01351-y)
Supplement: Supplementary file 2 — Supplementary file2 (DOCX 23 KB) [file 406_2021_1351_MOESM2_ESM.docx]

**Supplementary Table S2**

**Title:** Medium-term and peri-lockdown course of psychosocial burden during the ongoing Covid-19 pandemic: A longitudinal study on patients with pre-existing mental disorders

Claudia Bartels PhD^1^, Philipp Hessmann MD, MPH^1^, Ulrike Schmidt MD^1,2,3^, Jonathan Vogelgsang MD^1,4^, Mirjana Ruhleder PhD^1^, Alexander Kratzenberg MSc^1^, Marit Treptow MSc^1^, Thorgund Reh-Bergen MSc^1^, Mona Abdel-Hamid PhD^1,5^, Luisa Heß MSc^1^, Miriam Meiser MD^1^, Jörg Signerski-Krieger MD^1^, Katrin Radenbach MD^1^, Sarah Trost MD^1,6^, Björn H. Schott MD, PhD^1,7,8^, Jens Wiltfang MD^1,7,9^, Claus Wolff-Menzler MD, MA^1^*^&^*, Michael Belz PhD^1^*^&^***^*^**

^1^Department of Psychiatry and Psychotherapy, University Medical Center Goettingen, Germany

^2^Department of Psychiatry and Psychotherapy, University Hospital Bonn, Germany

^3^Maastricht University Medical Center, School for Mental Health and Neuroscience, Department of Psychiatry and Neuropsychology, Maastricht, The Netherlands

^4^McLean Hospital, Harvard Medical School, Translational Neuroscience Laboratory, Belmont, MA, USA

^5^Department of Psychiatry and Psychotherapy, University of Duisburg-Essen, LVR-Hospital Essen, Germany

^6^Geriatric Psychiatry, University Department of Geriatric Medicine FELIX PLATTER, Basel, Switzerland

^7^German Center for Neurodegenerative Diseases (DZNE), Goettingen, Germany

^8^Leibniz Institute for Neurobiology, Magdeburg, Germany

^9^Neurosciences and Signaling Group, Institute of Biomedicine (iBiMED), Department of Medical Sciences, University of Aveiro, Aveiro, Portugal

*^&^both authors contributed equally to the work as senior authors.*

***Corresponding author:** Claudia Bartels, Department of Psychiatry and Psychotherapy, University Medical Center Goettingen, von-Siebold-Str. 5, D-37075 Goettingen, Germany, [claudia.bartels@med.uni-goettingen.de](mailto:claudia.bartels@med.uni-goettingen.de), +49 551 3914397

**Supplementary Table S2** Comparison of general psychiatric symptoms and resilience between baseline (1^st^ lockdown) and follow-up (2^nd^ lockdown; repeated measures)

| *Goe-BSI items* | ***T_1_***  *M* ± SD | ***T_2_***  *M* ± SD | *p*^1^ |
| --- | --- | --- | --- |
| **(A) General psychiatric symptoms** | |  |  |
| 1. “I have become more vigilant than before the corona-crisis.” | 5.32 ± 3.23 | 5.44 ± 3.21 | .684 |
| 2. “Since the beginning of the crisis, I have spent more time on the internet or with media than before (except for home office).” | 4.33 ± 3.48 | 3.87 ± 3.54 | .137 |
| 3. “Due to the crisis, I have less drive to undertake and tackle things.” | 3.92 ± 3.09 | 3.59 ± 3.16 | .208 |
| 4. “I have been paying more attention to possible symptoms of illness in others since the crisis began.” | 3.61 ± 3.33 | 4.39 ± 3.41 | *.003^**^* |
| 5. “Since the beginning of the corona-crisis, I have been less physically active.” | 3.54 ± 3.56 | 3.91 ± 3.77 | .233 |
| 6. “I have been paying more attention to possible symptoms of illness in myself since the crisis began.” | 3.52 ± 3.20 | 4.47 ± 3.45 | *.001^***^* |
| 7. “I don’t enjoy things the way I used to since the beginning of the crisis.” | 3.10 ± 2.98 | 2.92 ± 3.05 | .497 |
| 8. “Feelings of anxiety have increased since the crisis began.” | 3.03 ± 3.10 | 3.12 ± 3.32 | .710 |
| 9. “Since the beginning of the crisis, I have increasingly withdrawn emotionally from others. (not meant: social distancing).” | 2.86 ± 3.05 | 2.64 ± 2.96 | .424 |
| 10. “Since the beginning of the crisis, I eat more or less than before.” | 2.74 ± 3.29 | 1.91 ± 2.93 | *.005^**^* |
| 11. “I feel more anger or I am more aggressive since the crisis began.” | 2.31 ± 2.70 | 2.13 ± 2.80 | .437 |
| 12. “My cognitive functions (orientation, comprehension, concentration, memory) have declined during the corona-crisis.” | 1.74 ± 2.84 | 1.78 ± 2.77 | .862 |
| 13. “Since the beginning of the corona-crisis, I have had more physical symptoms than before.” | 1.71 ± 2.72 | 1.35 ± 2.50 | .120 |
| 14. “Compared to the time before the crisis, I have more conflicts with other people.” | 1.47 ± 2.33 | 1.41 ± 2.44 | .770 |
| 15. “Since the beginning of the crisis, I have had greater craving for addictive substances (alcohol, illicit drugs) than before.” | 0.64 ± 1.83 | 0.59 ± 1.40 | .785 |
| 16. “Since the beginning of the crisis, I consume more alcohol or illicit drugs.” | 0.53 ± 1.67 | 0.42 ± 1.29 | .485 |
| 17. “Since the beginning of the crisis, I have felt watched and persecuted more often.” | 0.51 ± 1.46 | 0.77 ± 1.90 | .123 |
| 18. “Compared to the time before the crisis, physical or psychological violence in my partnership or family has increased.” | 0.44 ± 1.61 | 0.42 ± 1.58 | .846 |
| 19. “Since the beginning of the crisis, I have been taking more pills.” | 0.41 ± 1.59 | 0.51 ± 1.88 | .594 |
| 20. “Since the beginning of the crisis, I have felt more strongly that others are conspiring against me.” | 0.37 ± 1.17 | 0.46 ± 1.51 | .518 |
| 21. “Since the beginning of the crisis, I have more often special perceptions that others do not have (e.g. hearing voices, seeing people or things).” | 0.33 ± 1.32 | 0.28 ± 1.32 | .719 |
| 22. “Since the beginning of the crisis, I have been engaging in self-injurious behavior more frequently.” | 0.25 ± 1.18 | 0.49 ± 1.55 | *.049^*^* |
| **(B) Resilience** | |  |  |
| 1. “For me, some things have changed in a positive way during the pandemic.” | 4.52 ± 3.56 | 3.88 ± 3.70 | *.044^*^* |
| 2. “The pandemic also holds opportunities for me.” | 3.28 ± 3.52 | 2.66 ± 3.41 | *.037^*^* |

*Notes.* English translation of the Goe-BSI (Goettingen psychosocial Burden and Symptom Inventory) items for **(A)** general psychiatric symptoms and **(B)** resilience with means (*M*), and standard deviations (± SD) for baseline (*T_1_*: 1^st^ lockdown, March/April 2020) and follow-up (*T_2_*: 2^nd^ lockdown, November/December 2020). All items were answered on a Likert scale from 0 to 10 (0 = *“does not apply at all”* to 10 = *“fully applies”*). The mean values within the categories **(A)** and **(B)** are sorted by size in descending order for the baseline assessment. ^1^Uncorrected *p*-values (t-tests for repeated measures; *N* = 154 to 158; *df* = 153 to 157).
