# Supplementary material for: Medium-term and peri-lockdown course of psychosocial burden during the ongoing COVID-19 pandemic: a longitudinal study on patients with pre-existing mental disorders
Source: Eur Arch Psychiatry Clin Neurosci. 2021 Nov 25;272(5):757–71. doi: 10.1007/s00406-021-01351-y (PMC8614217; doi:10.1007/s00406-021-01351-y)
Supplement: Supplementary file 3 — Supplementary file3 (DOCX 18 KB) [file 406_2021_1351_MOESM3_ESM.docx]

**Supplementary Table S3**

**Title:** Medium-term and peri-lockdown course of psychosocial burden during the ongoing Covid-19 pandemic: A longitudinal study on patients with pre-existing mental disorders

Claudia Bartels PhD^1^, Philipp Hessmann MD, MPH^1^, Ulrike Schmidt MD^1,2,3^, Jonathan Vogelgsang MD^1,4^, Mirjana Ruhleder PhD^1^, Alexander Kratzenberg MSc^1^, Marit Treptow MSc^1^, Thorgund Reh-Bergen MSc^1^, Mona Abdel-Hamid PhD^1,5^, Luisa Heß MSc^1^, Miriam Meiser MD^1^, Jörg Signerski-Krieger MD^1^, Katrin Radenbach MD^1^, Sarah Trost MD^1,6^, Björn H. Schott MD, PhD^1,7,8^, Jens Wiltfang MD^1,7,9^, Claus Wolff-Menzler MD, MA^1^*^&^*, Michael Belz PhD^1^*^&^***^*^**

^1^Department of Psychiatry and Psychotherapy, University Medical Center Goettingen, Germany

^2^Department of Psychiatry and Psychotherapy, University Hospital Bonn, Germany

^3^Maastricht University Medical Center, School for Mental Health and Neuroscience, Department of Psychiatry and Neuropsychology, Maastricht, The Netherlands

^4^McLean Hospital, Harvard Medical School, Translational Neuroscience Laboratory, Belmont, MA, USA

^5^Department of Psychiatry and Psychotherapy, University of Duisburg-Essen, LVR-Hospital Essen, Germany

^6^Geriatric Psychiatry, University Department of Geriatric Medicine FELIX PLATTER, Basel, Switzerland

^7^German Center for Neurodegenerative Diseases (DZNE), Goettingen, Germany

^8^Leibniz Institute for Neurobiology, Magdeburg, Germany

^9^Neurosciences and Signaling Group, Institute of Biomedicine (iBiMED), Department of Medical Sciences, University of Aveiro, Aveiro, Portugal

*^&^both authors contributed equally to the work as senior authors.*

***Corresponding author:** Claudia Bartels, Department of Psychiatry and Psychotherapy, University Medical Center Goettingen, von-Siebold-Str. 5, D-37075 Goettingen, Germany, [claudia.bartels@med.uni-goettingen.de](mailto:claudia.bartels@med.uni-goettingen.de), +49 551 3914397

**Supplementary Table S3** Resilience strategies and activities (translated to English) with descriptive data

| *Goe-BSI items* | *M* ± SD |
| --- | --- |
| **“Particular helpful for my psychological well-being during the Corona crisis was … (0-10):”** | |
| 1. “Contact with friends/family” | 6.65 ± 3.11 |
| 2. “Hobbies” | 5.87 ± 3.49 |
| 3. “Contact with psychiatrist/psychotherapist” | 5.78 ± 3.56 |
| 4. “Fewer appointments” | 4.79 ± 3.60 |
| 5. “Being alone” | 4.54 ± 3.45 |
| 6. “Housework and gardening” | 4.53 ± 3.66 |
| 7. “Sports” | 4.38 ± 3.62 |
| 8. “Work” | 3.93 ± 4.07 |
| 9. “Occupation with information on the pandemic” | 3.19 ± 3.17 |
| 10. “Contact with other practitioners (except for psychiatrist/psychotherapist)” | 3.19 ± 3.79 |
| 11. “Discovering new digital media” | 3.11 ± 3.61 |
| 12. “Faith/spirituality” | 2.13 ± 3.05 |
| 13. “Other activities/strategies” | 1.69 ± 3.34 |

*Notes.* English translation of the Goe-BSI (Goettingen psychosocial Burden and Symptom Inventory) items on resilience strategies and activities; with means (*M*), and standard deviations (± SD). All items were answered on a Likert scale from 0 to 10 (0 = “*not helpful at all*” to 10 = “*completely helpful*”). The mean values are sorted by size in descending order (*N* = 150 to *N* = 157)
